# Magnetic Resonance Molecular Imaging of Extradomain B Fibronectin Improves Imaging of Pancreatic Cancer Tumor Xenografts

**DOI:** 10.3389/fonc.2020.586727

**Published:** 2020-10-30

**Authors:** Peter Qiao, Nadia R. Ayat, Amita Vaidya, Songqi Gao, Wenyu Sun, Samuel Chou, Zheng Han, Hannah Gilmore, Jordan M. Winter, Zheng-Rong Lu

**Affiliations:** ^1^Department of Biomedical Engineering, Case School of Engineering, Case Western Reserve University, Cleveland, OH, United States; ^2^Department of Radiology and Radiological Science, Johns Hopkins School of Medicine, Johns Hopkins University, Baltimore, MD, United States; ^3^Department of Pathology, University Hospitals Cleveland Medical Center, Cleveland, OH, United States; ^4^Case Comprehensive Cancer Center, Case Western Reserve School of Medicine, Case Western Reserve University, Cleveland, OH, United States; ^5^Department of Surgery, University Hospitals Cleveland Medical Center, Cleveland, OH, United States

**Keywords:** MRI, extradomain B fibronectin, pancreatic cancer, molecular imaging, tumor microenvironment

## Abstract

The survival of pancreatic cancer patients can be greatly improved if their disease is detected at an early, potentially curable stage. Magnetic resonance molecular imaging (MRMI) of oncoproteins is a promising strategy for accurate, early detection of the disease. Here, we test the hypothesis that MRMI of extradomain-B fibronectin (EDB-FN), an abundant oncoprotein in the tumor extracellular matrix, can overcome the stromal barriers of pancreatic cancer to facilitate effective molecular imaging and detection of small tumors. Specimens of normal, premalignant, and malignant human pancreatic tissues were stained with a peptide-fluorophore conjugate (ZD2-Cy5.5) to assess EDB-FN binding and expression. MRMI with ZD2-N3-Gd(HP-DO3A) (MT218) specific to EDB-FN and MRI with Gd(HP-DO3A) were performed in three murine models bearing human pancreatic cancer xenografts, including a Capan-1 flank model, a BxPC3-GFP-Luc and a PANC-1-GFP-Luc intrapancreatic xenograft model. Tumor enhancement of the contrast agents was analyzed and compared. Staining of human tissue samples with ZD2-Cy5.5 revealed high EDB-FN expression in pancreatic tumors, moderate expression in premalignant tissue, and little expression in normal tissue. MRMI with MT218 generated robust intratumoral contrast, clearly detected and delineated small tumors (smallest average size: 6.1 mm^2^), and out-performed conventional contrast enhanced MRI with Gd(HP-DO3A). Quantitative analysis of signal enhancement revealed that MT218 produced 2.7, 2.1, and 1.6 times greater contrast-to-noise ratio (CNR) than the clinical agent in the Capan-1 flank, BxPC3-GFP-Luc and PANC-1-GFP-Luc intrapancreatic models, respectively (*p* < 0.05). MRMI of the ECM oncoprotein EDB-FN with MT218 is able to generate superior contrast enhancement in small pancreatic tumors and provide accurate tumor delineation in animal models. Early, accurate detection and delineation of pancreatic cancer with high-resolution MRMI has the potential to guide timely treatment and significantly improve the long-term survival of pancreatic cancer patients.

## Introduction

Pancreatic cancer (PaCa) is responsible for a large and rapidly growing number of cancer deaths (1). PaCa prognosis remains poor, with a 5-year survival rate of merely 9% (2, 3). Patients often present with advanced-stage PaCa that has metastasized or cannot be surgically resected (1). Analysis of post-surgical outcomes suggests that the detection and removal of early-stage disease results in dramatically improved survival or disease cure (4). However, current strategies for PaCa diagnosis are not sensitive for early-stage disease. Contrast enhanced computed tomography (CE-CT) is the most commonly utilized for imaging of PaCa (5), but has difficulty for diagnosing small and potentially curable tumors, lymph node metastasis, and liver metastasis (5–8). Contrast enhanced magnetic resonance imaging presents superior soft tissue contrast and excellent spatial resolution, and is increasingly utilized in PaCa diagnosis (5, 9). However, the existing clinical contrast agents are not tumor-specific, and suffer from poor sensitivity in detecting small tumors (10). Development of tumor-specific contrast agents would improve intratumoral contrast agent accumulation and maximize the advantages of MRI for accurate detection and delineation of early-stage PaCa. There is an unmet need for safe and effective targeted MRI contrast agents that can detect early-stage PaCa.

Pancreatic cancer has a dense tumor stroma that impedes binding of molecular imaging agents that target cell-surface molecules and presents a formidable barrier for effective molecular imaging and cancer detection. Nevertheless, its unique extracellular matrix (ECM) molecular signature can be exploited to generate image contrast for precision molecular imaging and detection of small tumors (11–13). Extradomain B fibronectin (EDB-FN) is an oncofetal splice variant of fibronectin, and is reestablished in malignancy, but absent in most normal tissues (14–19). EDB-FN in the tumor ECM is readily accessible for specific binding of an imaging agent for effective molecular imaging of PaCa tumors. Its abundance in aggressive tumors allows rapid binding of sufficient targeted contrast agent to generate robust signal enhancement in magnetic resonance molecular imaging (MRMI). Therefore, EDB-FN is a promising target molecular imaging and early detection of PaCa with MRMI.

We previously developed an oligopeptide, TVRTSAD (ZD2), that binds with high specificity to EDB-FN (16). ZD2 targeted MRI contrast agents have been developed and tested for MRMI of EDB-FN in aggressive breast cancer and prostate cancer models (15, 16, 20). The targeted contrast agent ZD2-N3-Gd(HP-DO3A) (MT218) was developed by conjugating ZD2 peptide to a clinical macrocyclic contrast agent Gd(HP-DO3A) (16, 20). MT218 has a higher T_1_ relaxivity than Gd(HP-DO3A) and has demonstrated superior contrast enhancement in aggressive breast and prostate cancers (15, 16, 20–22). The binding of MT218 to abundant EDB-FN in PaCa tumor ECM has the potential to overcome the barriers of the dense tumor stroma to generate strong contrast enhancement to improve PaCa imaging with MRMI.

## Materials and Methods

### Institutional Review Board, Animal Study Approval, Disclosure of Conflict of Interest

The studies involving human participants were reviewed and approved by the University Hospitals Cleveland Medical Center Institutional Review Board, which determined that the studies performed with human tissues were not human subject research according to federal regulations under 45 CFR 46 or 21 CFR 56. All animal experiments were conducted in accordance to an animal protocol approved by the CWRU Institutional Animal Care and Use Committee. The targeted contrast agent MT218 was provided by Molecular Theranostics, LLC (Cleveland, OH). The authors had the full control of the data and information submitted for publication. Z.-R. Lu is one of the founders of Molecular Theranostics LLC and Motek Pharmaceuticals. S. Gao, Z. Han, and Z.-R. Lu have ownership interest in related patents. No potential conflicts of interest were disclosed by the other authors.

### Cells and Reagents

ZD2-N3-Gd(HP-DO3A) was obtained from Molecular Theranostics (Cleveland, OH). ProHance®, Gd(HP-DO3A), was purchased from Bracco Diagnostics (Monroe Township, NJ). ZD2-Cy5.5 was synthesized as previously described (16). Capan-1, BxPC3, and PANC-1 (ATCC, Manassas, VA) human PaCa cells were cultured in recommended culture media and conditions. Capan-1 cells are derived from the liver metastasis of a 40-year-old male with a well-differentiated primary tumor of ductal origin. BxPC3 cells are derived from the primary tumor of a 62-year-old female that was histologically classified as moderate to poorly differentiated. PANC-1 cells were derived from the primary tumor of a 56-year-old female that was histologically classified as poorly differentiated. BxPC3 and PANC-1 cells were transduced with a lentiviral vector (Amsbio, Cambridge, MA) for expression of green fluorescent protein (GFP) and luciferase.

### Relaxivity Measurements

The T_1_ and T_2_ values of MT218 and Gd(HP-DO3A) solutions were determined using a 3T MRS 3000 scanner (MR Solutions, Guildford, UK). Solutions of MT218 were prepared at concentrations of 0, 0.25, 0.5, 1, and 2 mM in DPBS buffer (Gibco, Gaithersburg, MD). T_1_ values were measured using an inversion recovery-fast low angle shot (IR-FLASH) sequence (τ = 10 ms, TE = 4 ms, FA = 8°, echoes/frames = 128, FOV = 40 mm × 40 mm, slice thickness = 2 mm, number of averages = 1, time delay = 4,000 ms, sample period = 200, matrix = 256 × 128). T_2_ values were obtained using a multi-echo multi-slice (MEMS) sequence (TE = 15 ms, repetition time = 15 ms, echoes = 10, FOV: 40 mm × 40 mm, slice thickness = 1 mm, number of averages = 1, matrix = 256 × 192). The r_1_ and r_2_ relaxivities were calculated from the slopes of 1/T_1_ and 1/T_2_ vs. Gd concentration plots, respectively.

### Binding Affinity Measurements

The binding affinity of MT218 was determined with microscale thermophoresis (MST) using a Nanotemper Monolith NT.115 instrument (NanoTemper Technologies, Munich, Germany). The EDB fragment of FN was expressed in *E. coli*, purified, and labeled with amine-reactive dye NT-647. MT218 was dissolved into assay buffer (50 mM PBS w/0.05% Tween-20). This solution was further diluted using assay buffer to give a series of MT218 working solutions. Each MT218 working solution was then mixed with a fixed concentration of fluorescently labeled EDB fragment. The mixtures of MT218 and EDB were then loaded into standard capillaries, and MST measurements were performed at 25°C using 20–80% light-emitting diode power and 40% infrared-laser power at varying concentrations of MT218.

### Animal Models

For flank xenograft models, 4 × 10^6^ Capan-1 cells suspended in 100 μL of a 6 mg/mL Matrigel (Corning, Corning, NY) solution was injected subcutaneously into the flank of 8-week-old female nu/nu mice. The tumor bearing mice were imaged after tumor volumes exceeded 300 mm^3^. For orthotopic intrapancreatic models, the pancreas of 4-week-old female nu/nu mice were surgically exposed via a ~10 mm incision in the left ventral abdomen and the application of gentle pressure to express the spleen and associated pancreatic tissue. 1 × 10^6^ BxPC3-GFP-Luc or PANC-1-GFP-Luc cells suspended in 100 μL of a Matrigel solution (6 mg/mL) was injected into the pancreatic tissue using an insulin syringe with a 31G needle. Any leakage of cell suspension was lavaged with DPBS twice to prevent intraabdominal seeding PaCa cells. The peritoneal fascia was closed with absorbable stitches, and the dermis closed with stainless steel wound clips. Prior to imaging, growth of tumor was verified by bioluminescence imaging with intraperitoneal injection of d-luciferin on IVIS Spectrum system (Perkin Elmer, Waltham, MA).

### ZD2-Cy5.5 Staining of Human Tissue

Tissue sections from PaCa patients (Case Comprehensive Cancer Center, Cleveland, OH) were deparaffinized with xylene, ethanol, and washed with water. Blocking was performed with 10% goat serum (Invitrogen, Carlsbad, CA) in PBS with 0.1% Tween 20 (PBS-T) for 30 min and incubated with 500 nM ZD2-Cy5.5 in PBS-T for 1 h at 37°C. Following three washes with PBS-T, the sections were mounted using Fluoroshield mounting medium (Abcam, Cambridge, UK). Images were acquired on a FV1000 (Olympus, Waltham, MA) confocal microscope using pre-programmed emission and excitation filters for Cy5.5 (excitation: 635 nm; emission: 693 nm) and DAPI (excitation: 405 nm; emission: 461 nm), using a 10 × objective lens. All tissue samples were imaged at the same laser power and sensor gain. Images were generated from single-channel grayscale images using FIJI software.

### Western Blotting Analysis of Cells and Tissue

Capan-1, BxPC3, and PANC-1 cells and tissues were lysed in RIPA buffer supplemented with cOmplete protease inhibitor cocktail (Roche, Basel, Switzerland). Tissue samples were further homogenized with a rotor-stator homogenizer (IKA, Wilmington, NC). Lysates were centrifuged and the supernatants were assayed for total protein concentration using the BCA protein assay (Thermo Fisher Scientific, Waltham, MA). Total protein (30 μg) was mixed in Laemli buffer (Bio-Rad, Hercules, CA) and boiled for 5 min. Samples were separated by SDS-PAGE (5–20%) and transferred onto nitrocellulose membrane (Cell Signaling Technologies, Danvers, MA). Primary antibodies used were anti-EDB-FN antibody BC-1 (1:500; Abcam, Cambridge, MA) diluted in 5% bovine serum albumin and anti-β-Actin antibody (1:1,000; Cell Signaling Technologies, Danvers, MA) diluted in 5% milk for cell lysates. G4 antibody (1:1,000; Absolute Antibody, Boston, MA) in 5% bovine serum albumin and anti-GAPDH antibody (1:1,000; Cell Signaling Technologies, Danvers, MA) in 5% milk were used for tissue lysates. Primary antibody incubation was performed overnight. Secondary anti-mouse-HRP (1:1,000; Cell Signaling Technologies, Danvers, MA) and anti-rabbit-HRP (1:1,000; Cell Signaling Technologies, Danvers, MA) antibodies were incubated at room temperature in 5% milk in TBS-T for 1 h with shaking. The stained membranes were developed with a SignalFire Plus ECL kit (Cell Signaling Technologies, Danvers, MA) and imaged using the ChemiDoc XRS+ system (Bio-Rad, Hercules, CA). Densiometric measurements were made with FIJI software.

### Immunohistochemical and Histochemical Staining of Mouse Tumor Tissue

PaCa tumor tissue and normal pancreatic tissue were collected from euthanized mice and fixed with 10% buffered formalin for 24 h. The samples were embedded in paraffin blocks and 5 μm sections were cut using a RM2235 microtome (Leica, Buffalo Grove, IL). Hematoxylin/eosin staining was performed under standard conditions. Antigen retrieval was performed at 125°°C for 30 s in pH 6.0 citrate buffer, followed by 3% H_2_O_2_ peroxidase block (8 min) and Rodent Block M (20 min) (Biocare Medical, Pacheco, CA). Anti-EDB-FN G4 monoclonal antibody (1:100) was incubated with tissue sections at RT for 1 h with shaking. Detection was performed with HRP Polymer detection solution (Biocare Medical, Pacheco, CA). Visualization was performed with 3,3′-diaminobenzidine for 5 min and counterstained with hematoxylin for 5 s. Images were acquired with an Bx61VS (Olympus, Waltham, MA) slide scanner and processed in OlyVIA software. Histological interpretation was performed by a board-certified pathologist.

For assessment of binding specificity of ZD2 peptide, flash frozen samples of tumor and normal tissues were collected from the euthanized tumor bearing mice and embedded in optimal cutting temperature media. The embedded samples were then immersed in liquid nitrogen for 5 min and transferred to −80°C for storage before cryosectioning. Tissue sections were blocked for 1 h in PBS containing 0.05% Tween-20 and 10% normal goat serum (Gibco, Gaithersburg, MD) and then stained with BC-1 primary monoclonal antibody (1:400) or ZD2-Cy5.5 (500 nM) at 4°C overnight or room temperature incubation for 2.5 h, respectively, and then washed with PBS-T buffer. Secondary Alexa Fluor 594 anti-rabbit was incubated with the BC-1 treated sections for 1 h at room temperature (1:1,000). For the blocking experiment, the tissue sections were first incubated with an excess of BC1 antibody at 4°C overnight, then washed three times with PBS-T. The sections were then incubated with ZD2-Cy5.5 at room temperature incubation for 2.5 h. The stained sections were washed with PBS-T buffer and images were acquired on an Olympus FV1000 (Waltham, MA) confocal microscope using pre-programmed emission and excitation filters for Cy5.5 (excitation: 635 nm; emission: 693 nm) and DAPI (excitation: 405 nm; emission: 461 nm), using a 10 × objective lens.

### Contrast-Enhanced MRI

Flank tumor-bearing mice were anesthetized and a tail vein catheter inserted. MR image acquisition was performed on a 7T Biospin (Bruker, Billerica, MA) preclinical small animal scanner with a volume radiofrequency coil. Axial images were obtained using a multi-spin multi-echo (MSME) MRI sequence (500 ms TR, 8.1 ms TE, 90° FA, 4.50 cm × 4.50 cm FOV, 1.50 mm slice thickness, 16 slices, 2 averages, 200 × 200 matrix, 0.0225 × 0.0225 cm/pixel resolution). Contrast agents were injected at a dose of 0.1 mmol/kg (100 μL) via the tail vein catheter, followed by a saline flush. Images of the tumors were acquired before contrast and at various time points after contrast administration. Gd(HP-DO3A) was used as a control.

Mice bearing intrapancreatic tumors were anesthetized and a tail vein catheter was inserted. Image acquisition was performed on a MR Solutions 3T MRS 3000 scanner with a mouse radiofrequency coil. Axial images were obtained using a Fast Spin Echo (FSE) MRI sequence (305 ms TR, 11 ms TE, 90° FA, 4 cm × 4 cm FOV, 1 mm slice thickness, 10 slices, echo train length = 4, echo spacing = 11 ms, 4 averages, 256 × 256 matrix, 0.0156 × 0.156 cm/pixel resolution) with respiratory gating. Images were acquired before contrast and at different time points after contrast injection at a dose of 0.1 mmol/kg (100 μL). For competitive binding experiments, a 5:1 molar ratio of free ZD2 peptide to MT218 was injected in mice bearing intrapancreatic BxPC3 tumor xenografts (*n* = 3) at a dose of 0.1 mmol MT218/kg (100 μL). Images obtained on the MR Solutions 3T scanner were normalized using the Surscalereader.exe (MR Solutions, Guildford, UK) program supplied.

Image files were exported and analyzed in Horus software. ROIs were drawn in the tumor, liver, kidney, and muscle. Muscle ROIs were taken from the muscle, with other structures (liver, kidney, tumor) identified after comparison with published images and comparison with anatomical landmarks or bioluminescent imaging. Pancreatic lesions were localized using the liver, kidney, and spleen as anatomical landmarks. Pancreatic tissue is located inferior and dorsal to the liver, ventral and superior to the kidney, and medial to the spleen (Supplementary Figure 1). Size of ROIs drawn for image analysis are summarized in Supplementary Table 1. The contrast-to-noise ratio of the tumor (CNR) was calculated using the following equation, where σ_*noise*_ is the standard deviation of intensities from an ROI drawn outside of the mouse body:

$$\begin{array}{l} CNR=\frac{{Mean\;Intensity}_{Tumor}-{Mean\;Intensity}_{Muscle}}{\sigma_{noise}}. \end{array}$$

The contrast to noise ratio of the liver (CNR) was calculated using the following equation:

$$\begin{array}{l} CNR=\frac{{Mean\;Intensity}_{Liver}-{Mean\;Intensity}_{Muscle}}{\sigma_{noise}} \end{array}$$

Image subtraction was performed on images adjusted to the same window and level in FIJI open-source software. Result of image subtraction was displayed using the RGB Rainbow color lookup table.

### Statistical Analysis

Image contrast was reported as fold enhancement of contrast to noise ratio of post-contrast images over pre-contrast images using Fold Enhancement = CNR_post_/CNR_pre_. Statistical analysis of CNR was performed in Minitab Express (Minitab Inc., State College, PA). One-way ANOVA with Fisher Individual Tests for Differences of Means *post-hoc* testing was performed to determine statistical significance between >2 means (*p* < 0.05). Student's T test was used to determine statistical significance between 2 means (*p* < 0.05).

## Results

### Binding Affinity and Relaxivities of MT218

**Figure 1:**
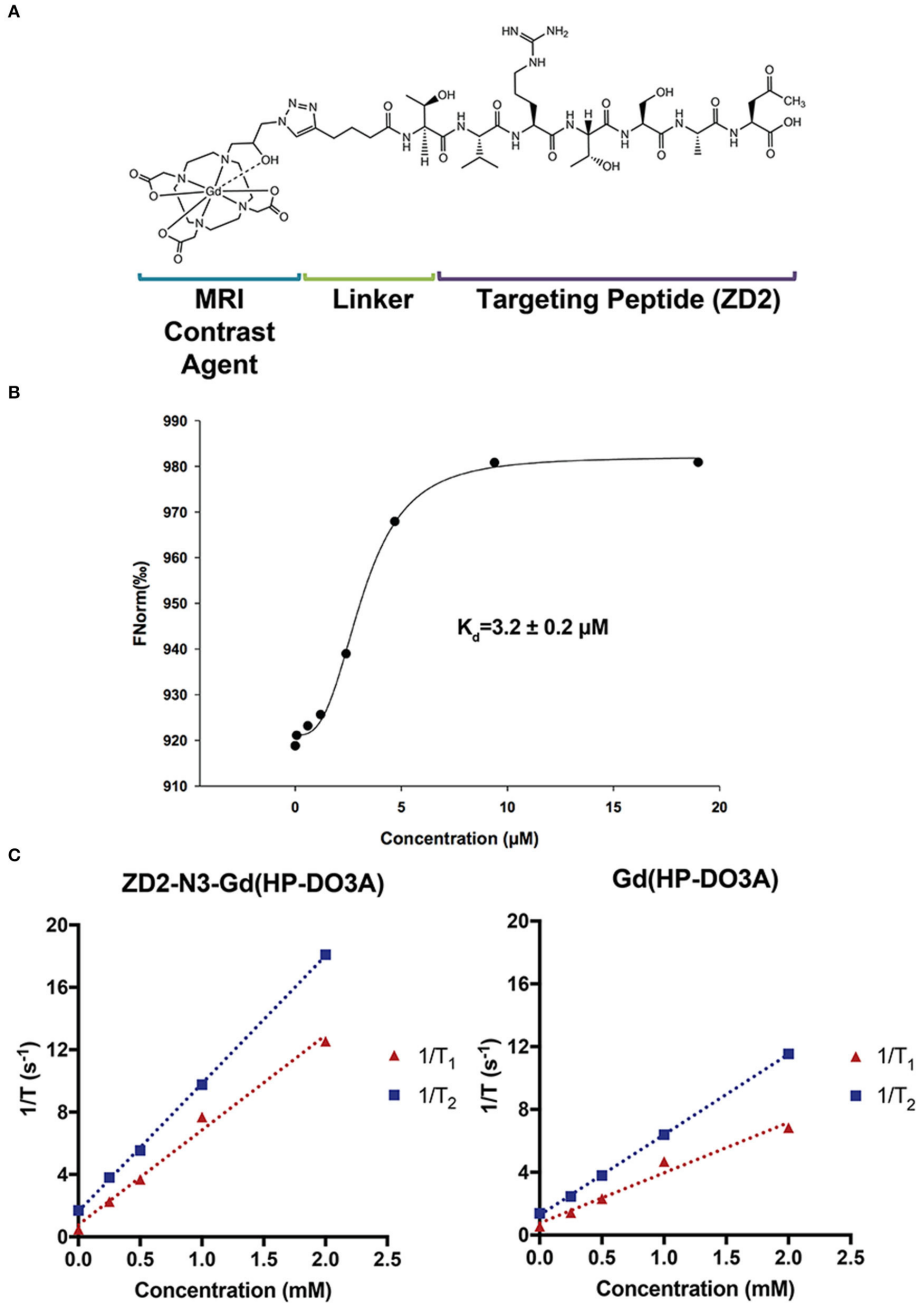
The structure **(A)**, binding affinity measurement **(B)**, and relaxivities **(C)** of the targeted contrast agent ZD2-N3-Gd(HP-DO3A) (MT218).

Figure 1 shows the chemical structure, binding affinity, and relaxivities of the targeted contrast agent MT218. MT218 is a small molecular conjugate of ZD2 peptide to a macrocyclic clinical MRI contrast agent Gd(HP-DO3A). The binding affinity of MT218 to EDB-FN was measured to be 3.2 ± 0.2 μM, which is similar to that of a previously targeted contrast agent ZD2-Gd(HP-DO3A) (23). The T_1_ and T_2_ relaxivities of MT218 were measured to be 6.07 ± 1.13 s^−1^ mM^−1^ and 8.20 ± 0.18 s^−1^ mM^−1^, respectively, at 3 T. T_1_ and T_2_ relaxivities of Gd(HP-DO3A) were measured to be 3.20 ± 0.96 s^−1^ mM^−1^ and 5.12 ± 0.18 s^−1^ mM^−1^, respectively, at 3 T. The targeted contrast agent has a nearly 2-fold of T_1_ relaxivity than the corresponding clinical agent at 3 T and should be more effective for T1-weighted contrast enhanced MRMI of solid tumors with elevated EDB-FN expression. The higher relaxivity of MT218 is possibly attributed to the relative rigid conjugation structure of the peptide to Gd(HP-DO3A).

### EDB-FN Expression in Human PaCa

**Figure 2:**
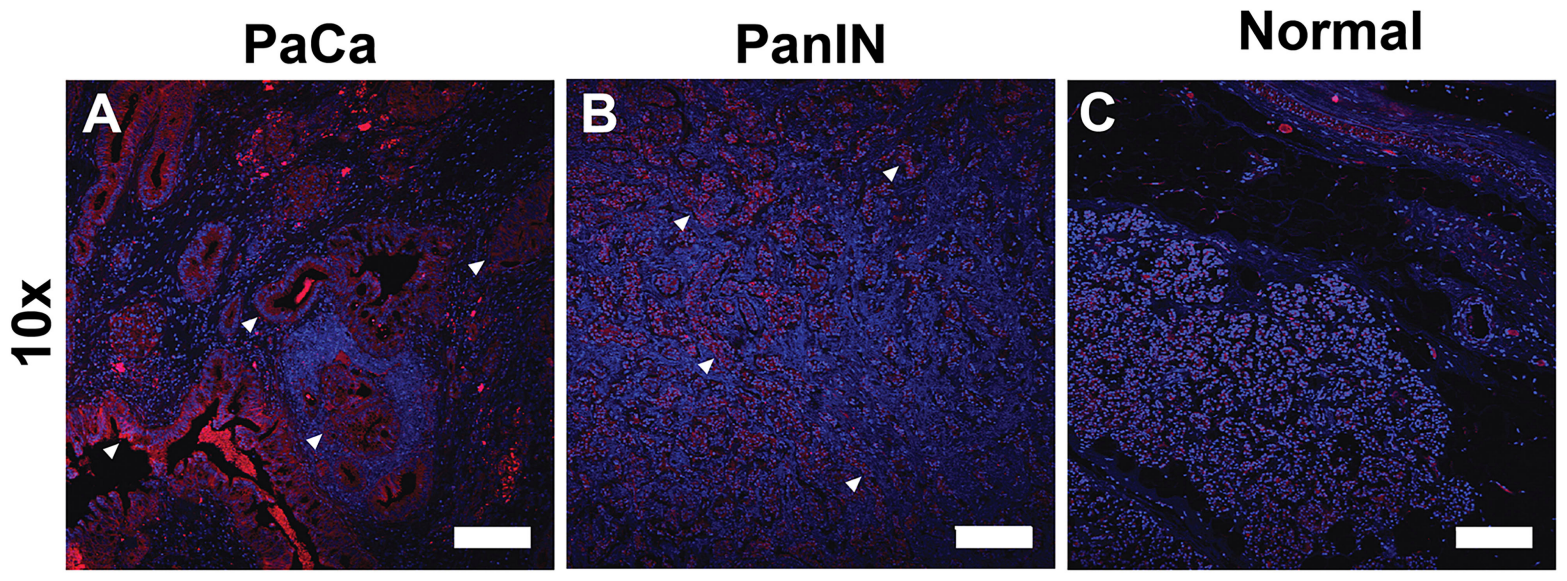
Fluorescence confocal microscopic images of extradomain B fibronectin expression taken with at 10× (Scale bar: 200 μm) magnification in human pancreatic cancer **(A)**, precancerous pancreatic intraepithelial neoplasia (PanIN) **(B)**, and normal pancreatic tissues **(C)** stained with ZD2-Cy5.5 (red) and DAPI (blue). Binding of the ZD2-Cy5.5 due to EDB-FN expression reveals regions of malignancy and potential malignancy (arrowheads).

Figure 2 shows the binding of ZD2 peptide to EDB-FN in human PaCa, pancreatic intraepithelial neoplasia (PanIN), and normal pancreatic tissue specimens with the targeted fluorescent probe ZD2-Cy5.5. ZD2-Cy5.5 binding was high in human PaCa tissue, moderate in precancerous PanIN tissue, and low in normal pancreatic tissue. The distribution of ZD2-Cy5.5 binding was heterogenous in malignant and premalignant tissues. Strong fluorescence intensity was seen in poorly organized ductal structures in the PaCa specimen, while intermediate staining was observed in the PanIN tissue with staining concentrated in the cell clusters with no luminal structure. The fluorescence intensity of ZD2-Cy5.5 binding in different human pancreatic tissues was indicative of high expression of EDB-FN in malignant PaCa tissue, intermediate expression in precancerous PanIN, and low expression in normal pancreas.

### EDB-FN Expression in Murine Models of PaCa

**Figure 3:**
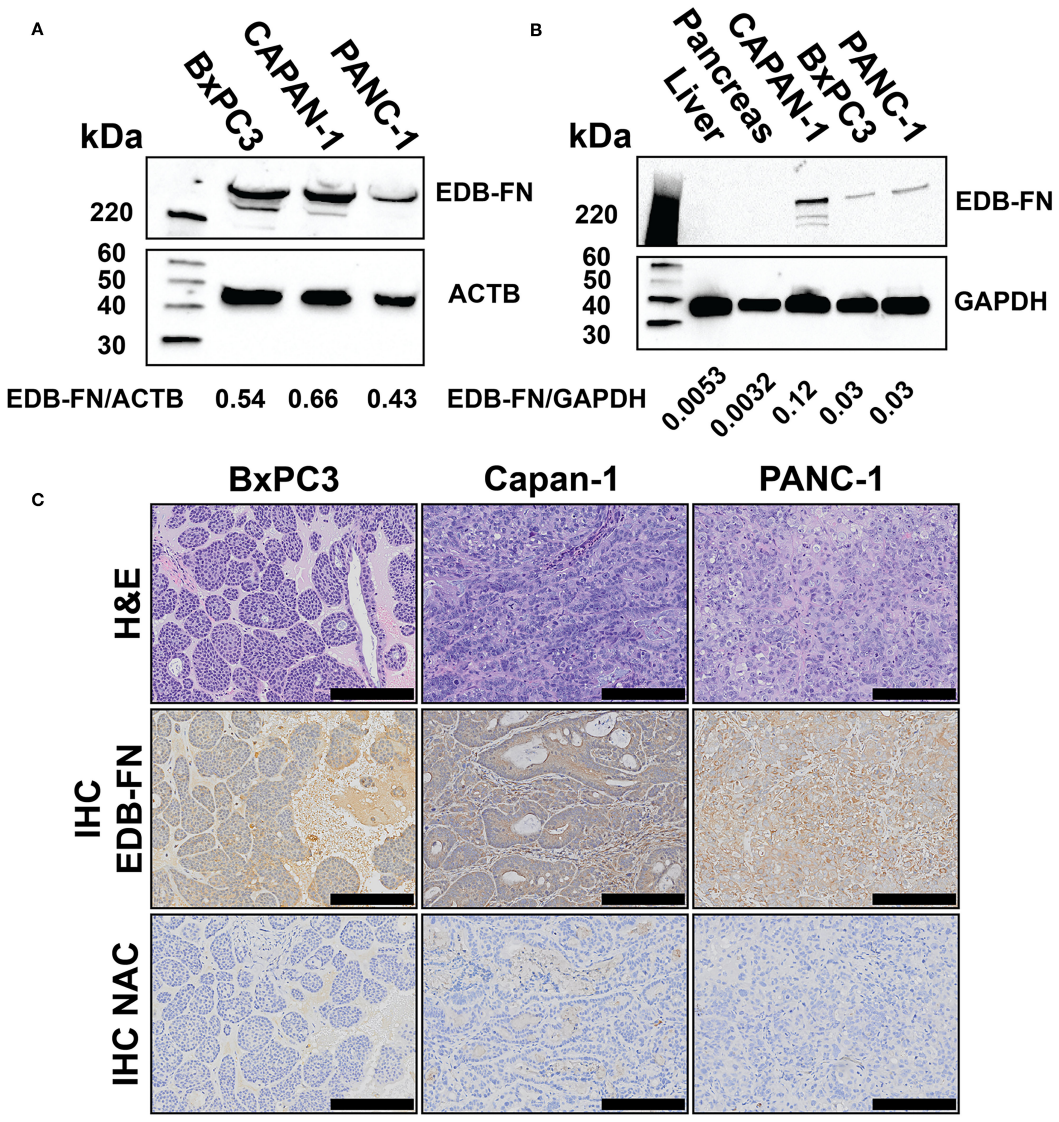
Western blots of extradomain B fibronectin (EDB-FN) in BxPC3-GFP-Luc (BxPC3), Capan-1, and PANC-1-GFP-Luc (PANC-1) human PaCa cells with densiometric quantification **(A)**, tumor xenografts derived from PaCa cells **(B)**, and immunohistochemical staining of EDB-FN in PaCa xenografts with and without (NAC) G4 anti-EDB-FN monoclonal antibody **(C)**, scale bar 200 μm, 10× magnification). Western blots of normal tissues and hematoxylin & eosin (H&E) staining of the tumor specimens were also shown as references.

The expression of EDB-FN was also evaluated in Capan-1, BxPC3-GFP-Luc, and PANC-1-GFP-Luc human PaCa cells and tumor xenografts derived from the cells. Western blotting with the EDB-FN specific G4 antibody revealed the expression of two 220+ kDa bands consistent with the size of EDB-FN protein in all three PaCa lines (Figure 3A). Western blotting of liver, pancreas, and tumor protein extracts from mice bearing the PaCa xenografts also showed the 220+ kDa band in all three PaCa models and not in the normal hepatic and pancreatic tissue, indicating elevated EDB-FN expression in the tumors and no expression in the normal tissues and organs (Figure 3B). Hematoxylin and eosin stained slides of the pancreatic tumor xenografts revealed large nodules of poorly differentiated carcinoma consistent with the appearance of PaCa (Figure 3C) (24). All tumors grew rapidly in the mice. Histological analysis exhibited different morphological patterns, but densely packed cells with low cytoplasmic to nuclear ratio in the tumors. Immunohistochemical staining with G4 anti-EDB-FN antibody exhibited strong staining throughout the tissue sections of all three PaCa models although the western blotting shows relatively different EDB-FN expression by the cells, while no staining was observed without the antibody (Figure 3C). Staining of flash frozen tissue sections with an EDB-FN specific monoclonal antibody BC-1 or ZD2-Cy5.5 revealed similar staining patterns for EDB-FN in BxPC3, Capan-1, and PANC-1 tissues. No staining was seen in normal pancreatic and muscle tissues, indicating no EDB-FN expression (Supplementary Figure 2). ZD2-Cy5.5 binding was blocked in the presence of the anti-EDB-FN antibody (Supplementary Figure 3). The results indicate that EDB-FN is overexpressed in human pancreatic cancer cells and their tumor xenografts in mice with no expression in normal tissues.

### MRMI of Pancreatic Cancer Flank Xenografts

**Figure 4:**
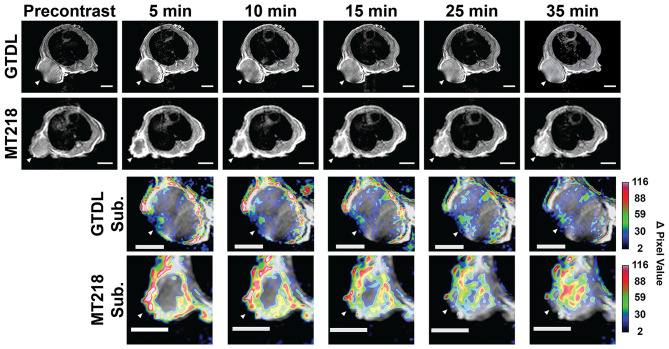
Representative T_1_-weighted 2D spin-echo MR images of mice bearing Capan-1 flank xenografts before and at different time points after injection of MT218 (*n* = 7) or Gd(HP-DO3A) (GTDL) (*n* = 5) with the corresponding subtraction images (post-contrast subtracted by pre-contrast) overlaid on pre-contrast images. Regions with large changes in tumor signal of the subtraction images are indicated in white/red, regions with small changes in tumor signal are indicated in black/blue. Tumors are marked by white arrowheads, scale bar: 5 mm.

The effectiveness of MRMI of EDB-FN with the targeted contrast agent MT218 in detecting PaCa was first assessed in mice bearing subcutaneous Capan-1 flank tumor xenografts. The clinical agent Gd(HP-DO3A) was used as a control at the same dose (0.1 mmol/kg). Stronger contrast enhancement was observed in the periphery of the tumors 5 min after injection with MT218 than with Gd(HP-DO3A) (Figure 4). The contrast enhancement in the tumor core then gradually increased in the mice injected with MT218 (Figure 4). The mice administered with Gd(HP-DO3A) did not exhibit significant tumor core enhancement. Subtraction of the pre-contrast images from the post-contrast images at different time points of the same mice illustrated the strong initial enhancement in the tumor periphery at the early time points (5–15 min post-contrast) and then robust tumor contrast enhancement throughout the tumors at later time points (25–35 min post-contrast) after MT218 administration (Figure 4). In comparison, the subtraction images of the mice administered Gd(HP-DO3A) produced modest peripheral contrast enhancement at early time points post-injection, and little to no intratumoral contrast at all time points. The results demonstrate that MT218 is effective in producing robust signal enhancement throughout the tumors for effective molecular imaging and detection of PaCa with MRMI.

### MRMI of Intrapancreatic Tumor Xenografts

**Figure 5:**
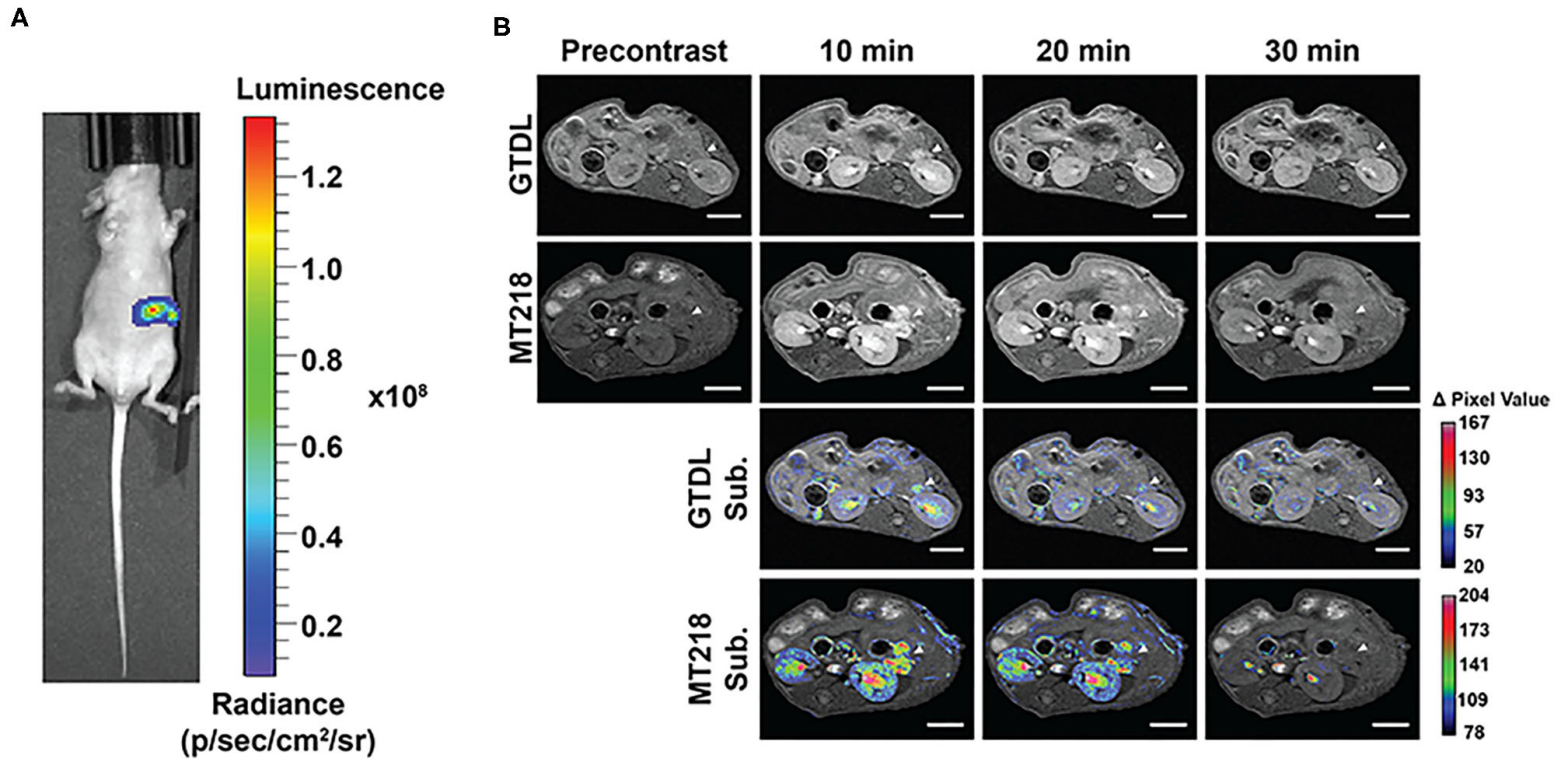
Bioluminescence images of mice implanted with BxPC3-GFP-Luc intrapancreatic tumors **(A)**. Representative T_1_-weighted 2D spin-echo MR images of intrapancreatic tumors before and at different time points after injection of MT218 or Gd(HP-DO3A) (GTDL) at 0.1 mmol/kg and the corresponding subtraction image overlaid on pre-contrast images (*n* = 3). Scale bar: 5 mm **(B)**. Arrowheads point to the intrapancreatic BxPC3-GFP-Luc PaCa tumors.

The effectiveness of MRMI with MT218 was further investigated in mice bearing intrapancreatic human PaCa tumor xenografts. Bioluminescence imaging verified the presence of luciferase-labeled BxPC3-GFP-Luc tumor xenografts in the left upper abdomen of three mice implanted with tumor cells (Figure 5A). MT218 MRMI of tumor bearing mice clearly delineated small lesions with strong contrast enhancement within 10 min of MT218 administration (Figure 5B). Tumor contrast enhancement then decreased at 20 min and returned to background levels 30 min after MT218 injection. The clinical agent Gd(HP-DO3A) generated modest contrast enhancement in the tumors at 10 min post-injection and the signal enhancement reduced to background level 20 min after Gd(HP-DO3A) injection (Figure 5B). Subtraction of the pre-contrast images from the post-contrast images further demonstrated strong enhancement and clear delineation of the intrapancreatic tumors with the targeted contrast agent at 10 min post-injection in T_1_-weighted MR images, while Gd(HP-DO3A) produced less intratumoral signal enhancement (Figure 5B). To further demonstrate the specificity of MT218 for MRMI of PaCa, a competitive binding experiment was performed with co-administration of a 5-fold excess of free ZD2 peptide and MT218 in mice bearing intrapancreatic BxPC3 xenografts. The co-injection of the excess free peptide blocked the binding of the MT218 to the tumors, resulting in significant reduction of contrast enhancement in the tumor images as compared to the tumor-bearing mice injected with MT218 with free ZD2 peptide (Supplementary Figure 4).

**Figure 6:**
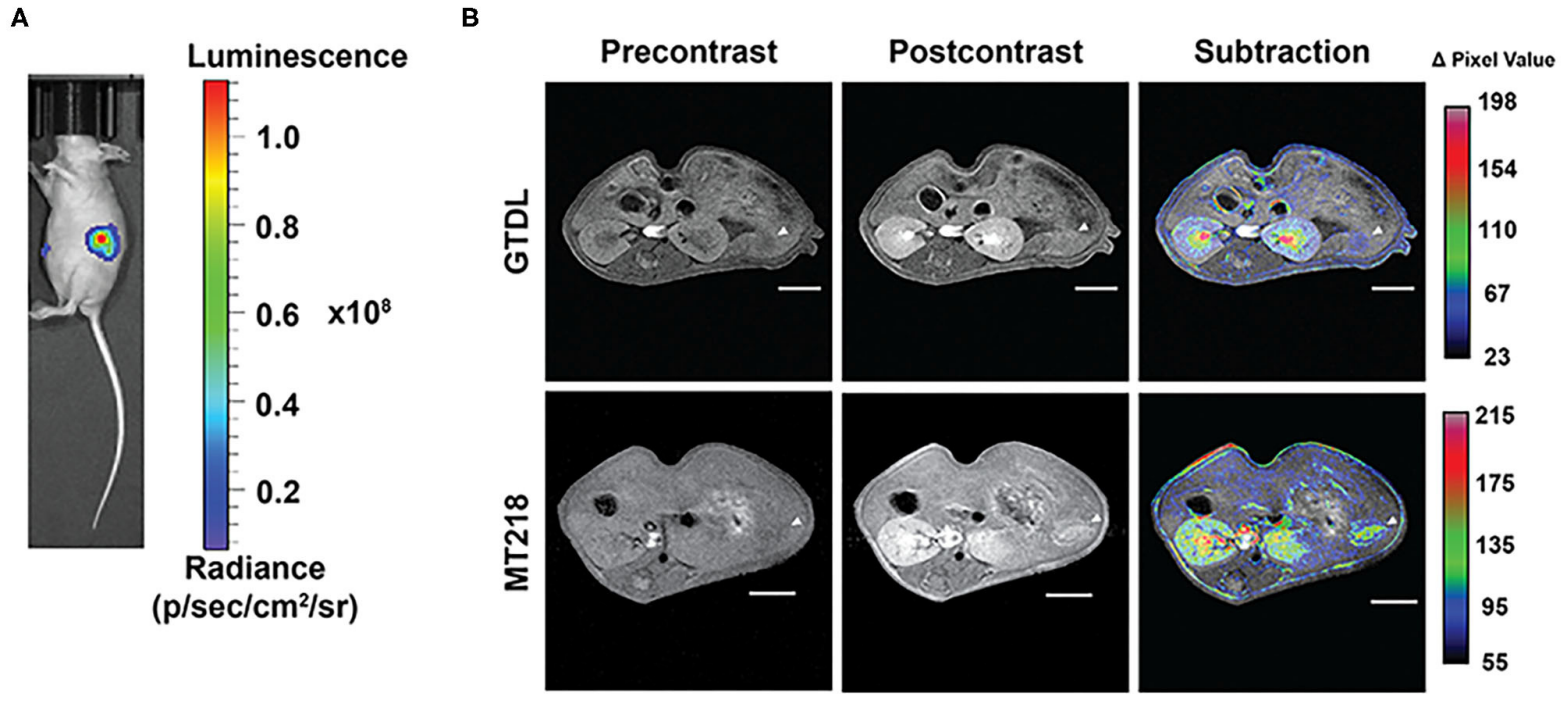
Bioluminescence images of the mice implanted with PANC-1-GFP-Luc intrapancreatic tumors **(A)** and representative T_1_-weighted 2D spin-echo MR images of the intrapancreatic tumors (*n* = 6) before and 10 min after injection of MT218 or Gd(HP-DO3A) (GTDL) at 0.1 mmol/kg with subtraction images overlaid on pre-contrast images **(B)**. Tumor locations are marked with arrowheads. Scale bar: 5 mm.

To determine whether the improved contrast enhancement in MRMI with MT218 is reproducible, MT218 MRMI was tested in another intrapancreatic PaCa model derived from PANC-1-GFP-Luc cells. Bioluminescence images of the mice bearing PANC-1-GFP-Luc intrapancreatic xenografts revealed substantial luciferase signal in the upper left abdominal region of cancer cell inoculation, indicating the tumor presence (Figure 6A). MT218 MRMI of tumor-bearing mice generated substantial contrast enhancement in tumor tissues and clearly delineated the tumors at 10 min post-injection. In contrast, Gd(HP-DO3A) did not produce significant tumor contrast enhancement and was not able to clearly identify the tumors (Figure 6B). Subtraction images of the pre-contrast images from the post-contrast images reveal substantial signal enhancement in the tumor with MT218, whereas little change was observed after Gd(HP-DO3A) administration (Figure 6B).

### Quantitative Analysis of ZD2 MRMI

**Figure 7:**
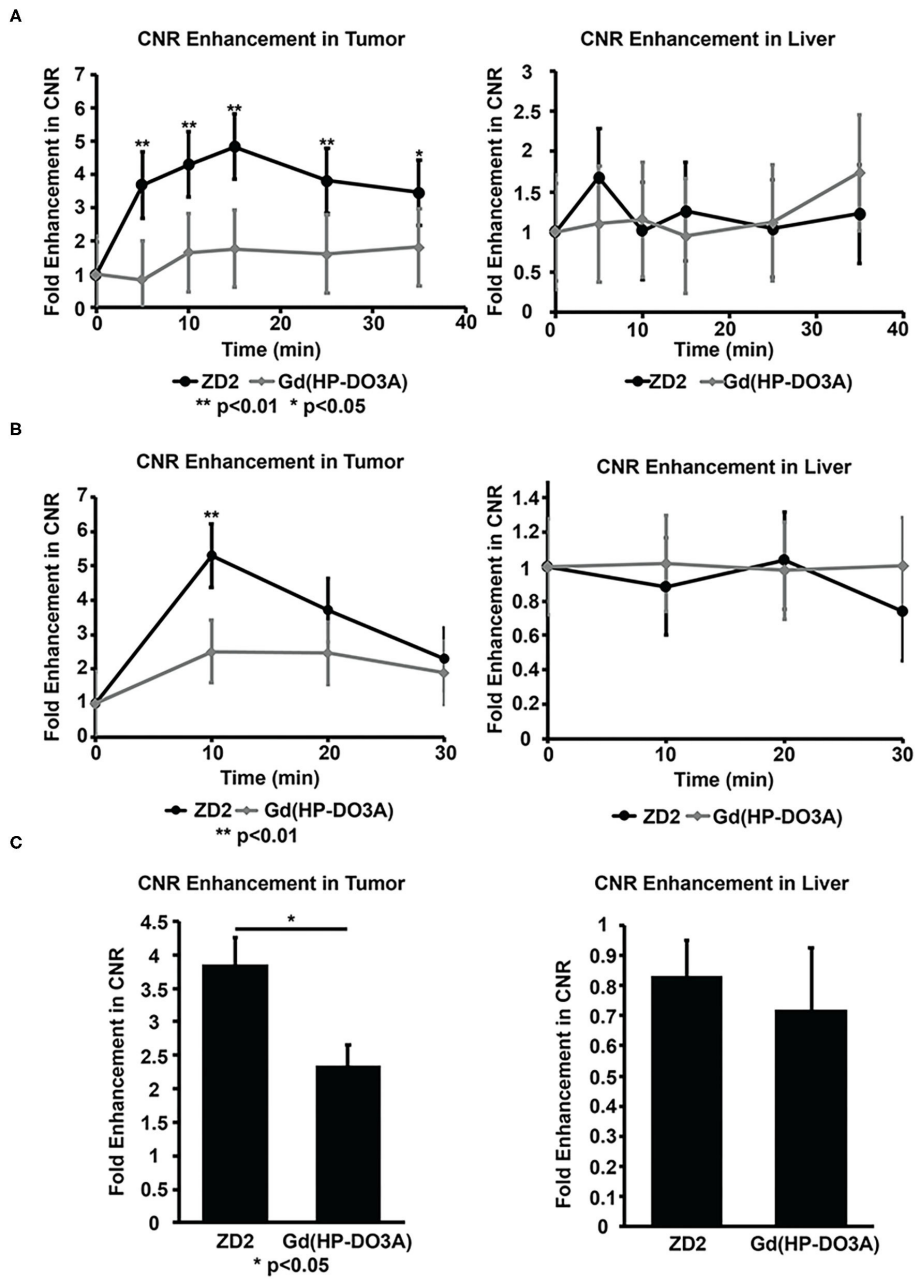
Contrast-to-noise ratios (CNR) of Capan-1 flank xenografts **(A)**, BxPC-GFP-Luc intrapancreatic tumors **(B)** and liver calculated from MRI images at different time points before and after injection of MT218 or Gd(HP-DO3A) (gadoteridol). CNR of the PANC-1-GFP-Luc intrapancreatic PaCa tumors calculated from MRI images taken before and after injection of MT218 and Gd(HP-DO3A) (gadoteridol) **(C)**. **p < 0.01, *p < 0.05.

MRMI tumor signal enhancement of MT218 was analyzed in comparison with the clinical agent. The signal enhancement in the liver was also analyzed to determine the potential non-specific contrast enhancement of MT218 in normal tissues. MT218 generated a maximum 4.8-fold increase of contrast to noise ratio (CNR) in the Capan-1 flank tumor xenografts at 15 min post-injection that maintained for at least 35 min (Figure 7A). The clinical agent Gd(HP-DO3A) produced an intratumoral CNR increase of 1.77-fold for the duration of the experiment, which was significantly less than MT218 (*p* < 0.01). No significant difference in CNR was observed in the liver (Figure 7A), kidney, and spleen (Supplementary Figure 5) of all tested mice between the two contrast agents (*p* > 0.05).

MT218 produced a maximum 5.3-fold CNR increase in the BxPC3-GFP-Luc intrapancreatic tumors at 10 min post-injection, while Gd(HP-DO3A) produced approximately 2.5-fold tumor CNR increase at 10 min, significantly less than that with MT218 (*p* < 0.01) (Figure 7B). The tumor CNR subsequently decayed for both agents. Similarly, MT218 produced approximately 3.9-fold intratumoral CNR increase in the PANC-1-GFP-Luc intrapancreatic tumor xenografts 10 min post-injection, whereas Gd(HP-DO3A) produced a 2.35-fold tumor CNR increase (Figure 7C). No difference in CNR was observed in the liver (Figures 7B,C) and kidneys (Supplementary Figure 5) of all tested mice between the two contrast agents (*p* > 0.05).

Co-injection of an excess of free ZD2 peptide with MT218 resulted in approximately 2.5-fold CNR increase in the BxPC3-GFP-Luc intrapancreatic model, which was similar to the CNR increase with Gd(HP-DO3A) (Figure 7B, Supplementary Figure 4). The CNR increase with MT218 in the presence of 5-fold free peptide was significantly lower than the CNR increase with MT218 without the free peptide (ca., 4.5-fold, *p* < 0.05). The co-injection of free ZD2 peptide inhibited the binding of MT218 and reduced tumor CNR to the level of non-specific accumulation of the agent similar to that of Gd(HP-DO3A). The results indicate the specific binding of MT218 to PaCa tumors.

## Discussion

The clinical use of magnetic resonance molecular imaging (MRMI) is desirable for precision cancer imaging as it combines the high soft tissue contrast and good spatial resolution of MRI with the ability to image and characterize molecular changes within tumors. A major challenge to clinical MRMI is to overcome the stromal barrier for sufficient binding of a targeted contrast agent to generate detectable contrast enhancement. Pancreatic cancer (PaCa) is highly fibrotic with a dense ECM, which limits the access of contrast agents to the inner tumor tissues (25). Many molecular imaging agents are bulky and bind to cell-surface targets that are difficult to reach. By exploiting oncospecific expression of extradomain B fibronectin (EDB-FN) in the tumor ECM as a target for MRMI, we found that the imaging of PaCa could be substantially improved.

We found that expression of EDB-FN could be detected in human PaCa and precancerous tissues with the EDB-FN specific fluorescent probe ZD2-Cy5.5, suggesting that MT218 MRMI may also be useful for characterizing premalignancy and malignancy. The expression of EDB-FN in PaCa and premalignancy is consistent with the observations of other groups (26). Interestingly, the expression of EDB-FN was highest in the Capan-1 cell line derived from a PaCa metastasis, suggesting that expression of EDB-FN may vary between primary tumors and metastasis. Nevertheless, all tumor models grew fast in mice and the IHC staining with G4 antibody showed similar EDB-FN expression in all tumors. The effectiveness of EDB-FN MRMI for detection of PaCa was demonstrated in mouse models of PaCa using the molecular imaging agent ZD2-N3-Gd(HP-DO3A) (MT218). MT218 binds to EDB-FN with micromolar affinity, consistent with previous reports (16, 23). Furthermore, the relaxivity of MT218 is higher than that of Gd(HP-DO3A). MT218 generates substantially greater image contrast-to-noise (CNR) compared to the clinical agent Gd(HP-DO3A) in Capan-1 flank (273% CNR of control, *p* < 0.05), BxPC3-GFP-Luc (212% CNR of control, *p* < 0.05) and PANC-1-GFP-Luc intrapancreatic (164% CNR of control, *p* < 0.05) murine models of PaCa due to its specific tumor binding and high T_1_ relaxivity. Although the relative CNR increase with MT218 seems correlated to the expression level of EDB-FN determined by the western blotting of the tumors cells, all three tumors had similar high CNR increase in the range of 4–5-folds. Further quantitative assessment is needed to accurately correlate the MRMI signal with the expression of EDB-FN in different tumors of similar aggressiveness.

The specific binding of MT218 to the EDB-FN enriched PaCa was validated by the decreased tumor CNR due to the disruption of MT218 binding by an excess of free ZD2 peptide in the intrapancreatic model. Time course MRMI images reveal that MT218 is able to bind to EDB-FN deep within the tumor core, which was not observed in imaging studies utilizing the untargeted clinical contrast agent Gd(HP-DO3A). Moreover, the small size (1,442 Da), moderate binding affinity, and hydrophilicity of MT218 contribute to its mobility for diffusion and binding within the tumor core while maintaining low background noise. Tumor nodules with area as small as 6.13 mm^2^ were better visualized with MT218, suggesting that MT218 has the potential for accurate early detection of PaCa that may improve early detection to initiate timely treatment for better patient survival. This is consistent with the performance of other ZD2 targeted imaging agent, which was able to detect small metastases using MRMI (27). This is critical for timely initiation of therapy and improved therapeutic outcomes. It has been reported that detection and surgical removal of PaCa tumors <10 mm in size will greatly improve the long-term survival and quality of life of PaCa patients (28).

Molecular imaging of EDB-FN in cancer has been previously investigated with antibodies labeled with radioisotopes for PET imaging. Anti-EDN-FN antibody BC-1 was labeled with ^125^I and tested in mice bearing U87 brain tumor and SKMel28 melanoma xenografts (29). Although the antibody is highly specific to EDB-FN and was found to bind to the EDB-FN rich tumors, its long-term retention in the circulation due to its large size generated significant background noise and affected the quality of specific tumor imaging. Nanobodies with a smaller size were recently developed for molecular imaging of EDB-FN in various types of tumor models. A ^64^Cu-labeled nanobody probe (^64^Cu-NJB2) demonstrated specific uptake and effective detection of PaCa and premalignant lesions in a mouse model utilizing PET/CT (26). ZD2 peptide has also been labeled with ^68^Ga as a PET probe for molecular imaging of EDB-FN. The ZD2 targeted probe provided sensitive and specific molecular imaging of EDB-FN in PaCa (30). Compared to PET, MRMI is advantageous for the delineation of small PaCa with high resolution and soft tissue contrast, which is valuable for treatment planning. The clinical implementation of PET/MRI provides a unique approach for molecular imaging of PaCa by targeting EDB-FN with PET probes and MRI contrast agents.

Other approaches to EDB-FN MRMI are also being investigated. Dextran-based chemical exchange saturation transfer (CEST) MRI has also utilized the ZD2 peptide to image PaCa (31). The Dextran-ZD2 conjugate generated detectable intratumoral signal in a flank model of PaCa over 45 min, supporting the hypothesis that MRMI of EDB-FN provides diagnostic value. However, CEST faces several challenges to translation, including lower signal to noise ratio at clinical field strengths, low sensitivity, and high doses. So far, gadolinium based contrast agents, especially the macrocyclic agents, are considered as the safe and effective contrast agents for clinical cancer MRI.

Although we have shown the promise of MRMI of EDB-FN in PaCa imaging, there are limitations to this study. Comprehensive investigation of EDB-FN expression in a large human PaCa population is needed to validate it as a biomarker for PaCa. It is possible that EDB-FN expression may vary greatly in human PaCa. However, the high degree of correlation between our results and another small-scale study of EDB-FN expression in human PaCa suggests that this is unlikely to be the case (26). The models utilized for this study may not fully recapitulate the tumor microenvironment of human PaCa due to the impairment of immune function in nu/nu mice. Further studies of MT218 are currently being conducted in immunocompetent and spontaneous models of PaCa. There is also a need for further epidemiological investigation of relevant risk factors and screening tools that would work in concert with MRMI to detect early pancreatic cancer. MRMI can improve the detection of early stage PaCa, especially for high risk populations, but is not an ideal screening tool in the general population. It is our belief that clinical translation of MRMI with ZD2-N3-Gd(HP-DO3A) will facilitate the rapid development of general population screening tools that can identify high risk patients who may benefit from MRMI detection of early stage PaCa.

In summary, this study investigates the overexpression of EDB-FN in human PanIN, PaCa specimens, and in murine models of PaCa, and demonstrates the effectiveness of MRMI of EDB-FN with a small molecular targeted MRI contrast agent MT218. MRMI with MT218 generates superior contrast enhancement and clearly delineates small PaCa tumors. Minimal non-specific signal enhancement was observed in the hepatic tissue. MRMI with MT218 has the potential for surveillance of precancerous pancreatic lesions and for precision detection and delineation of small pancreatic cancer. Clinical translation of MRMI with MT218 has the promise to addresses the unmet clinical need for a highly specific imaging technology to detect early-stage pancreatic cancer, and to impact a variety of aspects of clinical management of pancreatic cancer, including screening the high-risk populations, diagnosis, treatment decision making, and post-treatment surveillance and monitoring.

## Data Availability Statement

The raw data supporting the conclusions of this article will be made available by the authors, without undue reservation.

## Ethics Statement

The studies involving human participants were reviewed and approved by University Hospitals Cleveland Medical Center Institutional Review Board. Written informed consent for participation was not required for this study in accordance with the national legislation and the institutional requirements. The animal study was reviewed and approved by CWRU Institutional Animal Care and Use Committee. Written informed consent was not obtained from the individual(s) for the publication of any potentially identifiable images or data included in this article.

## Author Contributions

The conceptual design of the research was devised by Z-RL, PQ, and ZH. Experimental execution of all aspects of the research was done by PQ. PQ and NA performed MRMI. AV and SC assisted with cell assays and animal dissections. SG and WS performed relaxivity and binding affinity measurements. PQ, NA, and JW performed image analysis. HG reviewed the histology slides. The manuscript was written and edited by PQ and Z-RL, and reviewed by all authors.

## Conflict of Interest

Z-RL is one of the founders of Molecular Theranostics LLC and Motek Pharmaceuticals. SG, ZH, and Z-RL have ownership interest in related patents. The remaining authors declare that the research was conducted in the absence of any commercial or financial relationships that could be construed as a potential conflict of interest.

## References

[1] Siegel Rebecca L Miller Kimberly D Jemal A Cancer statistics, 2018. CA Cancer J Clin. (2018) 68:7–30. 10.3322/caac.21442 29313949

[2] Chiaravalli M Reni M O'Reilly EM. Pancreatic ductal adenocarcinoma: state-of-the-art 2017 and new therapeutic strategies. Cancer Treat Rev. (2017) 60:32–43. 10.1016/j.ctrv.2017.08.007 28869888

[3] Enzler T Bates S. Clinical trials in pancreatic cancer: a long slog. Oncologist. (2017) 22:1424–6. 10.1634/theoncologist.2017-0453 28982802 PMC5728038

[4] Katz MHG Wang H Fleming JB Sun CC Hwang RF Wolff RA. Long-term survival after multidisciplinary management of resected pancreatic adenocarcinoma. Ann Surg Oncol. (2009) 16:836–47. 10.1245/s10434-008-0295-2 19194760 PMC3066077

[5] Lee ES Lee JM Imaging diagnosis of pancreatic cancer: a state-of-the-art review. World J Gastroenterol. (2014) 20:7864–77. 10.3748/wjg.v20.i24.7864 24976723 PMC4069314

[6] Kim JH Park SH Yu ES Kim M-H Kim J Byun JH. Visually isoattenuating pancreatic adenocarcinoma at dynamic-enhanced ct: frequency, clinical and pathologic characteristics, and diagnosis at imaging examinations. Radiology. (2010) 257:87–96. 10.1148/radiol.10100015 20697118

[7] Yoon SH Lee JM Cho JY Lee KB Kim JE Moon SK. Small (≤ 20 mm) pancreatic adenocarcinomas: analysis of enhancement patterns and secondary signs with multiphasic multidetector CT. Radiology. (2011) 259:442–52. 10.1148/radiol.11101133 21406627

[8] Zins M Matos C Cassinotto C. Pancreatic adenocarcinoma staging in the era of preoperative chemotherapy and radiation therapy. Radiology. (2018) 287:374–90. 10.1148/radiol.2018171670 29668413

[9] Burk KS Lo GC Gee MS Sahani DV. Imaging and Screening of Pancreatic Cancer. Radiol Clin North Am. (2017) 55:1223–34. 10.1016/j.rcl.2017.06.006 28991562

[10] Hussain T Nguyen QT. Molecular imaging for cancer diagnosis and surgery. Adv Drug Deliv Rev. (2014) 66:90–100. 10.1016/j.addr.2013.09.007 24064465 PMC4464660

[11] Procacci P Moscheni C Sartori P Sommariva M Gagliano N. Tumor?stroma cross-talk in human pancreatic ductal adenocarcinoma: a focus on the effect of the extracellular matrix on tumor cell phenotype and invasive potential. Cells. (2018) 7:158. 10.3390/cells7100158 30301152 PMC6209911

[12] Weniger M Honselmann KC Liss AS. The extracellular matrix and pancreatic cancer: a complex relationship. Cancers. (2018) 10:316. 10.3390/cancers10090316 30200666 PMC6162452

[13] Han Z Lu Z-R. Targeting fibronectin for cancer imaging and therapy. J Mater Chem B. (2017) 5:639–54. 10.1039/C6TB02008A 29270293 PMC5733799

[14] Schwarzbauer JE DeSimone DW. Fibronectins, their fibrillogenesis, and *in vivo* functions. Cold Spring Harb Perspect Biol. (2011) 3:a005041. 10.1101/cshperspect.a005041 21576254 PMC3119908

[15] Han Z Wu X Roelle S Chen C Schiemann WP Lu Z-R. Targeted gadofullerene for sensitive magnetic resonance imaging and risk-stratification of breast cancer. Nat Commun. (2017) 8:692. 10.1038/s41467-017-00741-y 28947734 PMC5612990

[16] Han Z Zhou Z Shi X Wang J Wu X Sun D. EDB fibronectin specific peptide for prostate cancer targeting. Bioconjug Chem. (2015) 26:830–8. 10.1021/acs.bioconjchem.5b00178 25848940

[17] Inufusa H Nakamura M Adachi T Nakatani Y Shindo K Yasutomi M. Localization of oncofetal and normal fibronectin in colorectal cancer. Correlation with histologic grade, liver metastasis, and prognosis. Cancer. (1995) 75:280–8. 10.1002/1097-0142(19950615)75:12<2802::aid-cncr2820751204>3.0.co;2-o 7773930

[18] Arnold SA Loomans HA Ketova T Andl CD Clark PE Zijlstra A. Urinary oncofetal ED-A fibronectin correlates with poor prognosis in patients with bladder cancer. Clini Exp Metastasis. (2016) 33:29–44. 10.1007/s10585-015-9754-x 26456754 PMC4742427

[19] Midulla M Verma R Pignatelli M Ritter MA Courtenay-Luck NS George AJT. Source of oncofetal ED-B-containing fibronectin: implications of production by both tumor and endothelial cells. Cancer Res. (2000) 60:164. 10646869

[20] Ayat NR Qin J-C Cheng H Roelle S Gao S Li Y. Optimization of ZD2 peptide targeted Gd(HP-DO3A) for detection and risk-stratification of prostate cancer with MRI. ACS Med Chem Lett. (2018) 9:730–5. 10.1021/acsmedchemlett.8b00172 30034609 PMC6047029

[21] Ayat NR Vaidya A Yeung GA Buford MN Hall RC Qiao PL. Effective MR molecular imaging of triple negative breast cancer with an EDB-fibronectin-specific contrast agent at reduced doses. Front Oncol. (2019) 9:1351. 10.3389/fonc.2019.01351 31850230 PMC6901824

[22] Li Y Han Z Roelle S DeSanto A Sabatelle R Schur R. Synthesis and assessment of peptide Gd–DOTA conjugates targeting extradomain B fibronectin for magnetic resonance molecular imaging of prostate cancer. Mol Pharm. (2017) 14:3906–15. 10.1021/acs.molpharmaceut.7b00619 28976766 PMC6026531

[23] Han Z Li Y Roelle S Zhou Z Liu Y Sabatelle R. Targeted contrast agent specific to an oncoprotein in tumor microenvironment with the potential for detection and risk stratification of prostate cancer with MRI. Bioconjug Chem. (2017) 28:1031–40. 10.1021/acs.bioconjchem.6b00719 28201871 PMC6075728

[24] Niederhuber JE Armitage JO Doroshow JH Kastan MB Tepper JE Abeloff MD Abeloff's Clinical Oncology. 5th ed. Philadelphia, PA: Saunders/Elsevier (2014).

[25] Cao J Pickup S Clendenin C Blouw B Choi H Kang D. Dynamic contrast-enhanced MRI detects responses to stroma-directed therapy in mouse models of pancreatic ductal adenocarcinoma. Clin Cancer Res. (2019) 25:2314. 10.1158/1078-0432.CCR-18-2276 30587546 PMC6445712

[26] Jailkhani N Ingram JR Rashidian M Rickelt S Tian C Mak H. Noninvasive imaging of tumor progression, metastasis, and fibrosis using a nanobody targeting the extracellular matrix. Proc Natl Acad Sci USA. (2019) 116:14181. 10.1073/pnas.1817442116 31068469 PMC6628802

[27] Han Z Cheng H Parvani JG Zhou Z Lu Z-R. Magnetic resonance molecular imaging of metastatic breast cancer by targeting extradomain-B fibronectin in the tumor microenvironment. Magn Reson Med. (2018) 79:3135–43. 10.1002/mrm.26976 29082597 PMC5882210

[28] Tsunoda T Yamamoto Y Kimoto M Imai H Iwamoto S Kawasaki S. Staging and treatment for patients with pancreatic cancer. How small is an early pancreatic cancer? J Hepatobiliary Pancreat Surg. (1998) 5:128–32. 10.1007/s005340050022 9745077

[29] Mariani G Lasku A Balza E Gaggero B Motta C Di Luca L. Tumor targeting potential of the monoclonal antibody BC-1 against oncofetal fibronectin in nude mice bearing human tumor implants. Cancer. (1997) 80(12 Suppl.):2378–84. 9406686 10.1002/(sici)1097-0142(19971215)80:12+<2378::aid-cncr7>3.3.co;2-0

[30] Gao S Qin J Sergeeva O Sergeev M Qiao P Roelle S. Synthesis and assessment of ZD2-((68)Ga-NOTA) specific to extradomain B fibronectin in tumor microenvironment for PET imaging of pancreatic cancer. Am J Nucl Med Mol Imaging. (2019) 9:216–29. 31772820 PMC6872477

[31] Han Z Zhang S Fujiwara K Zhang J Li Y Liu J. Extradomain-B fibronectin-targeted dextran-based chemical exchange saturation transfer magnetic resonance imaging probe for detecting pancreatic cancer. Bioconjug Chem. (2019) 30:1425–33. 10.1021/acs.bioconjchem.9b00161 30938983 PMC6896991

