# Supplementary material for: Magnetic Resonance Molecular Imaging of Extradomain B Fibronectin Improves Imaging of Pancreatic Cancer Tumor Xenografts
Source: Front Oncol. 2020 Oct 30;10:586727. doi: 10.3389/fonc.2020.586727 (PMC7661967; doi:10.3389/fonc.2020.586727)
Supplement: Supplementary file 1 [file Data_Sheet_1.docx]

Supplementary Material

# Supplementary Figures and Tables

## Supplementary Figures


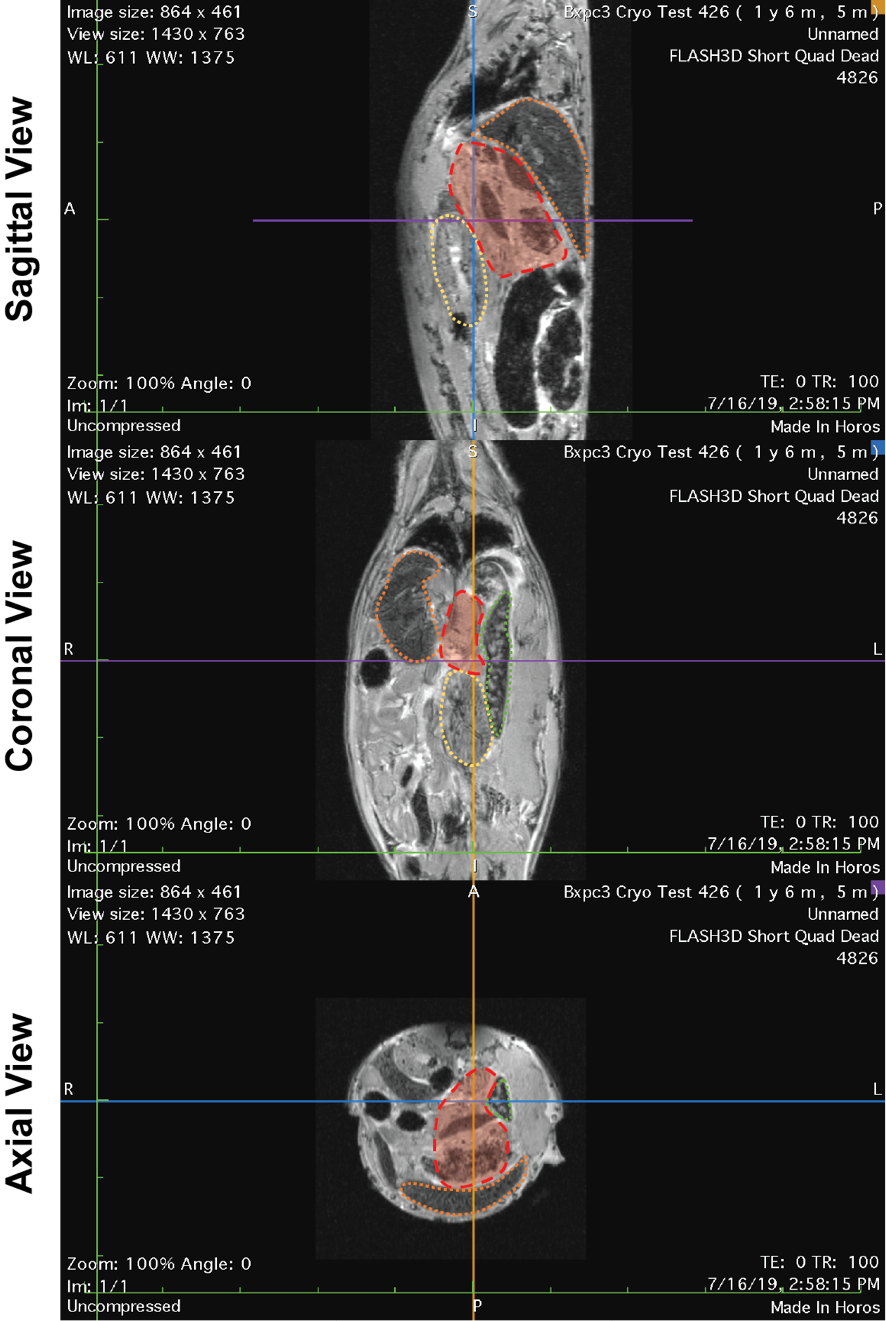


**Supplemental Figure S1:** Multiplanar reconstruction of FLASH 3D MRI data from a euthanized mouse not used in MRMI studies. Region containing mouse pancreatic tissue (Red dashed outline & shading) is located inferior and dorsal to the liver (orange dotted outline), superior and ventral to the kidney (yellow dotted outline), and medial to the spleen (green dotted outline).

**
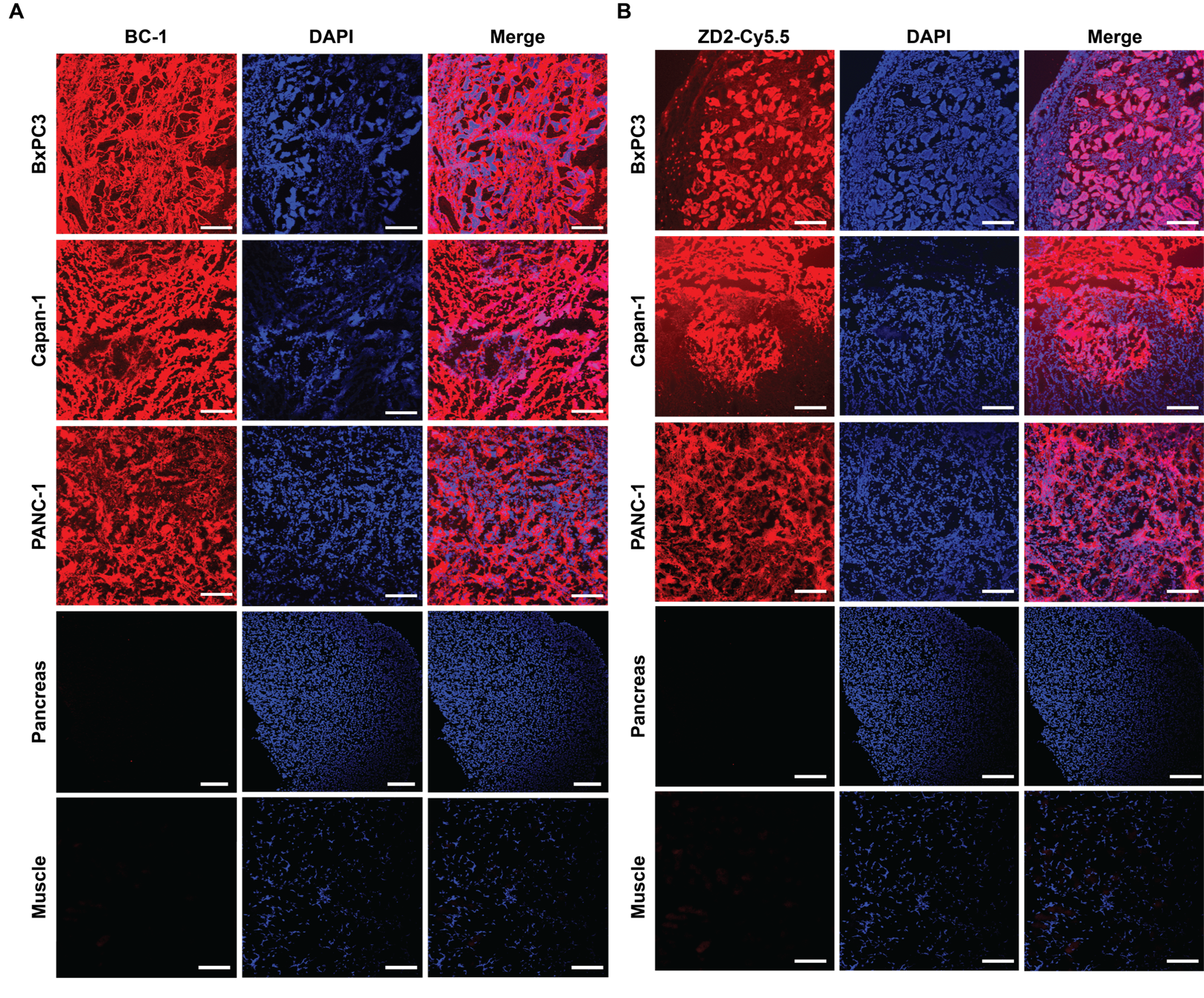
**

**Supplemental Figure S2:** Immunostaining of frozen tissue sections from tumor and normal tissues stained with anti-EDB-FN antibody BC-1 **(A)** or ZD2-Cy5.5 fluorescent dye conjugate **(B)**. Staining with BC-1 and ZD2-Cy5.5 is high in all three tumor tissues, but undetectable in normal pancreatic and muscle tissues.


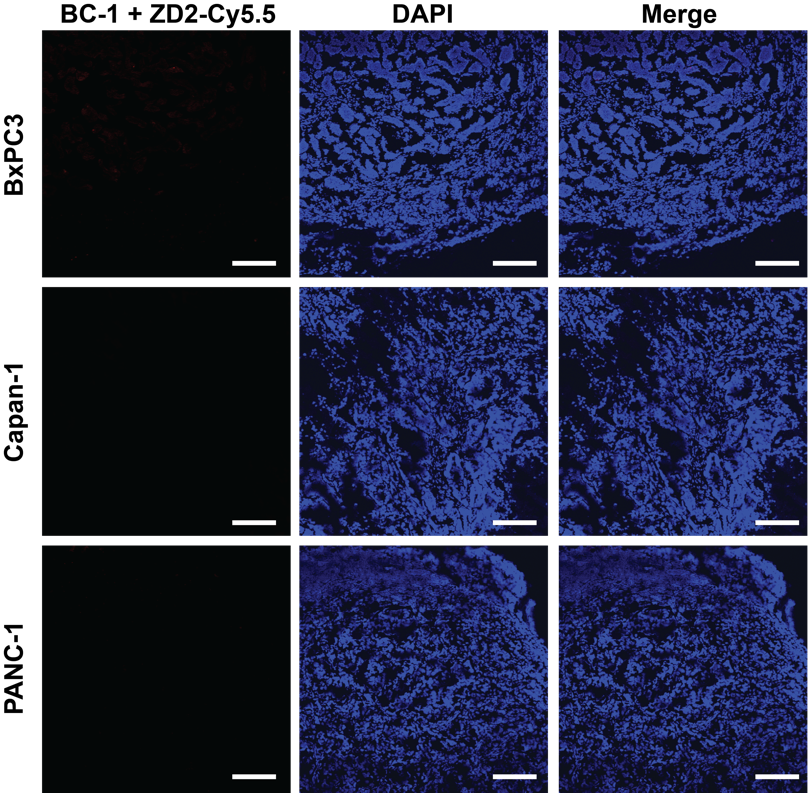


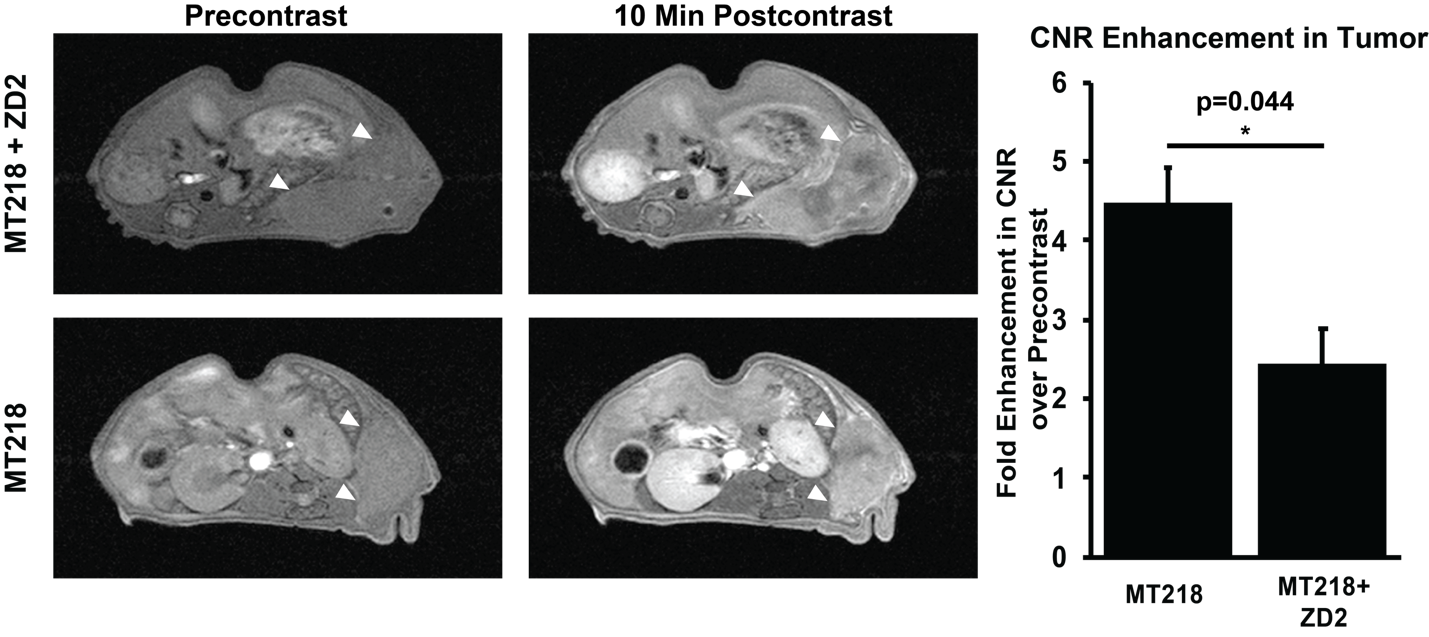
**Supplemental Figure S3:** Competitive binding of anti-EDB-FN antibody BC-1 and ZD2 fluorescent probe conjugate ZD2-Cy5.5 in flash frozen tumor tissues. No fluorescent signal is detected from ZD2-Cy5.5 after blocking of tissues with BC-1.

**Supplemental Figure S4:** MRMI images and contrast-to-noise (CNR) ratios of mice injected with MT218 or coinjected with MT218 and free ZD2 targeting peptide in a 1:5 molar ratio (MT218 + ZD2). A significant decrease in tumor CNR was observed (n = 3, p<0.05), with CNR of the MT218 + ZD2 group producing a CNR similar to that observed with Gd(HP-DO3A). Tumor locations are marked with arrowheads.

**
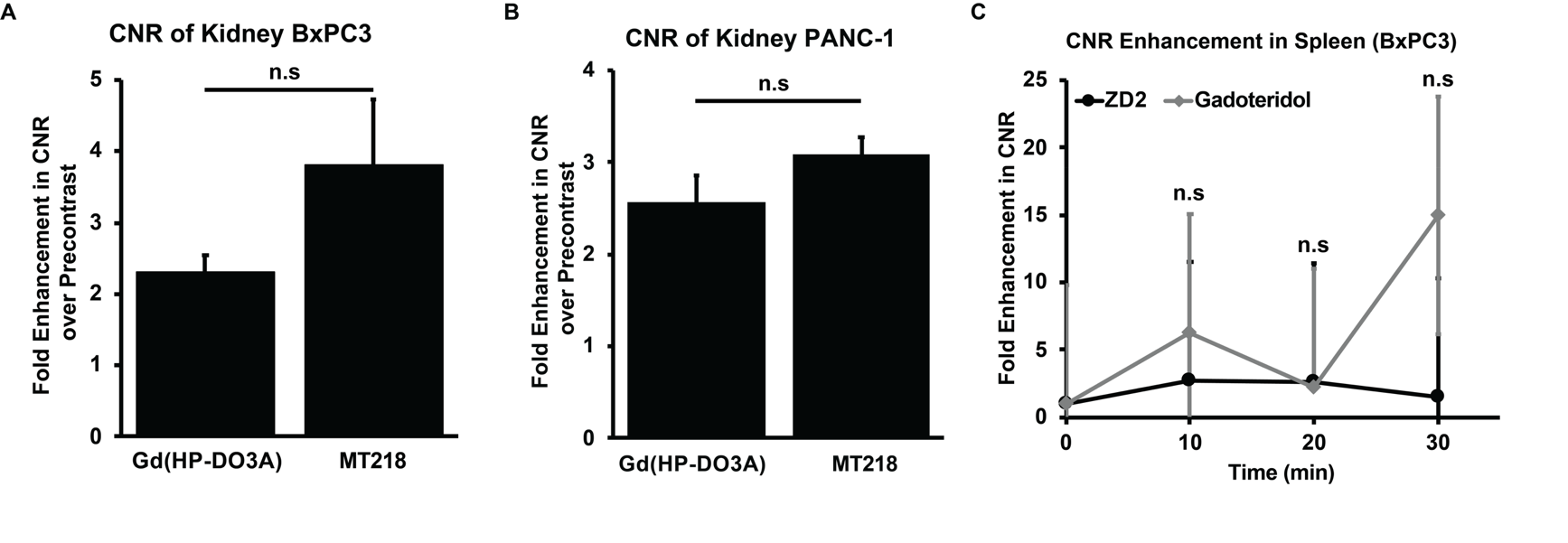
Supplemental Figure S5:** Contrast-to-noise (CNR) ratios of the kidneys of mice bering BxPC3-GFP-Luc intrapancreatic tumors (n = 3) **(A)** and PANC-1-GFP-Luc intrapancreatic tumors (n=6) **(B)**. No significant (n.s, p>0.05) difference in kidney CNR was detected, between the Gd(HP-DO3A) and MT218 groups, indicating that intravenous injections were uniformly patent. CNR of the spleen in the BxPC3-GPF-Luc (n=3) **(C)** intrapancreatic model. No significant (n.s, p>0.05) difference in spleen CNR was observed between the Gd(HP-DO3A) and MT218 groups.

| **Imaging Study** | **Tumor ROI (mm^3^)** | **Liver ROI (mm^3^)** | **Muscle ROI (mm^3^)** | **Noise ROI (mm^3^)** | **Kidney ROI (mm^3^)** |
| --- | --- | --- | --- | --- | --- |
| **Capan-1 + MT218** | 51.17 ± 6.79 | 10.53 ± 1.77 | 5.44 ± 1.00 | 18.99 ± 5.58 | N/A |
| **Capan-1 + Gadoteridol** | 80.32 ± 6.41 | 12.92 ± 1.36 | 8.08 ± 1.25 | 15.23 ± 2.25 | N/A |
| **BxPC3 + MT218** | 6.13 ± 2.94 | 6.32 ± 2.19 | 4.6 ± 0.47 | 10.23 ± 0.98 | 23.34 ± 0.92 |
| **BxPC3 + Gadoteridol** | 8.11 ± 1.56 | 7.45 ± 2.70 | 5.01 ± 0.34 | 9.21 ± 0.98 | 25.22 ± 1.08 |
| **PANC-1 + MT218** | 8.24 ± 1.74 | 15.13 ± 1.42 | 7.17 ± 0.74 | 15.39 ± 1.76 | 21.93 ± 2.18 |
| **PANC-1 + Gadoteridol** | 8.23 ± 1.21 | 13.94 ± 1.64 | 7.06 ± 0.64 | 17.42 ± 2.24 | 24.14 ± 1.67 |

**Supplemental Table S1:** Mean area (mm^3^) and standard error of ROIs used in CNR analysis. The kidney was not in the field of view of Capan-1 MRI images and no Kidney ROIs were measured.
